# Simultaneous injection‐aspiration technique of air/fluid exchange for in‐office treatment of post‐operative vitreous cavity hemorrhage

**DOI:** 10.1186/s40942-021-00282-z

**Published:** 2021-02-16

**Authors:** Riley N. Sanders, Sami H. Uwaydat

**Affiliations:** 1 Department of Ophthalmology, Jones Eye Institute, 4301 West Markham, Mail Slot 523, Little Rock, AR 72205 USA; 2 Department of Ophthalmology, Jones Eye Institute, 4301 West Markham, Mail slot 526, Little Rock, AR 72205 USA

**Keywords:** Proliferative diabetic retinopathy, Pars plana vitrectomy, Air fluid exchange

## Abstract

**Background:**

Post-operative vitreous cavity hemorrhage following pars plana vitrectomy is common. In-office drainage of the hemorrhage may be an option for some patients.

**Technique:**

A new method for office-based air fluid exchange is described. A 30-gauge needle with a 10-mm syringe filled with sterile air is inserted 3.5-mm posterior to the limbus in the superotemporal quadrant. A second 30-gauge needle is inserted 3.5 mm from the limbus at 6 o’clock and connected to an empty 10-mm syringe with intravenous catheter tubing. The plunger of the air-filled syringe is pushed while the plunger of the empty syringe is pulled, so that the rate of fluid aspiration matches the rate of air injection.

**Discussion:**

The method approximates conditions in pars plana vitrectomy, with balanced infusion and aspiration. Displaced vitreous cavity contents are collected in the aspiration syringe. The procedure is also cost effective.

**Conclusion:**

The simultaneous syringe method is an easy, safe, and effective way of clearing post-operative vitreous cavity hemorrhage.

## Introduction

Post-operative vitreous cavity hemorrhage (POVCH) is a common occurrence after pars plana vitrectomy (PPV), prolonging visual recovery. POVCH can be classified in two main forms: (1) early/persistent bleeding, usually from oozing of dissected neovascular tissue and sclerostomy sites, and (2) late/recurrent, often from anterior fibrovascular ingrowth (FVI) or entry site neovascularization [1]. The reported incidence of early POVCH varies widely from 5 to 80%, but with modern small gauge vitrectomy, the rate is probably around 1/3 [1]. Intraoperative strategies during PPV to reduce incidence of POVCH include adequate removal of posterior vitreous, vitrectomy and panretinal photocoagulation using deep scleral indentation, and intraocular tamponade [1]. There is some evidence that intravitreal anti-vascular endothelial growth factor (VEGF) drugs may reduce the incidence of early POVCH [2]. Over 90% of POVCH will clear within 5–6 weeks [3], but when significant hemorrhage persists longer, additional procedures may be required to clear the blood [3]. FVI and anterior tractional membranes should be considered and searched for to aid in forming a surgical plan. If FVI is suspected on examination or ultrasonography, repeat PPV with more aggressive laser or cryoablation of the anterior vitreous base may be necessary [4]. In the absence of FVI, vitreous cavity lavage or air-fluid exchange (AFX) is an option for select patients.

Methods for in-office fluid-fluid exchange [5] and air-fluid exchange [6–9] have been described. These involve either a one-needle open system [7, 9]; or a two-needle open system [5]. A different approach can be performed using a two-needle closed system utilizing a non-dependent air-injection needle and dependent fluid-aspiration needle. The efficacy, risk profile, and cost analysis of this technique has previously been reported [8]. The purpose of this paper is to describe in detail the simultaneous syringe technique for performing office based AFX and compare to the other techniques.

## Technique

Patients are considered for in-office AFX if at 4–6 weeks after PPV, they still have significant non-clearing vitreous hemorrhage. If a patient is monocular, AFX may be considered as early as the first post-operative week. B-scan ultrasonography is performed to exclude a retinal detachment, and to rule out persistent or new tractional membranes.

**Fig. 1:**
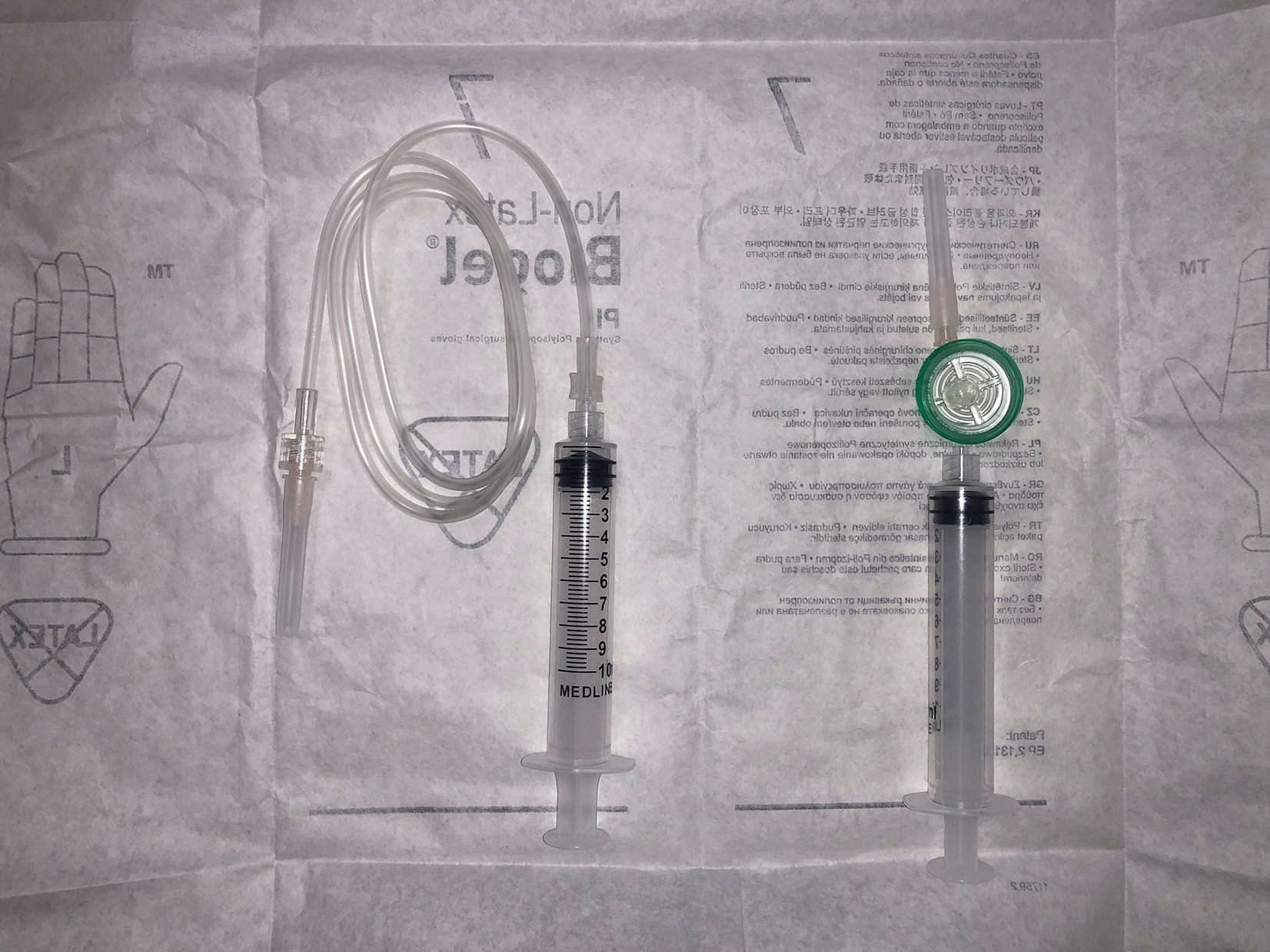
Materials for air fluid exchange. Two 10 cm^3^ syringes; one syringe attached by intravenous catheter tubing to a 30-gauge needle, and a second 30-gauge needle on a syringe filled with filtered air

Materials for the procedure are gathered (Fig. 1: Photography of setup). The patient is placed in a sitting position, and the examination chair is elevated so that the patient’s eye is almost at the level of the surgeon’s shoulder. After topical anesthesia and subconjunctival 1% lidocaine are given in the superotemporal and inferior quadrants, the eye is prepped with a 5% povidone iodine solution and a lid speculum is placed.

Using sterile techniques, a 10 mL syringe is filled with about 7 mL of filtered air. A half-inch 30-gauge needle is then attached and inserted 3.5 millimeters (mm) posterior to the supratemporal limbus (nondependent position) through the pars plana into the vitreous cavity. A second 30-gauge needle, attached to a short length of intravenous catheter tubing that is connected to an empty 10 mL syringe, is then inserted through the pars plana 3.5 mm posterior to the limbus at 6 o’clock (dependent position). Injection of air through the nondependent syringe is performed by the surgeon, while the plunger of the dependent syringe is slowly withdrawn by an assistant, so that rate of aspiration approximates the rate of injection. The procedure is continued until air is observed in the dependent syringe. The dependent needle is then removed. Additional air is injected or withdrawn to adjust intraocular pressure with the surgeon digitally checking the globe. The non-dependent needle is then removed, which concludes the procedure (Additional file 1: video). The eye is then irrigated with saline, and few drops of topical antibiotics are applied.

Vision is then confirmed to be at least hand motion, and intraocular pressure is checked with a Tono-Pen ® (Reichert ®) 10 min after the procedure, prior to the patient leaving the clinic.

## Discussion

Office-based air fluid exchange is a good option for certain patients with POVCH after PPV, and the simultaneous syringe method reported here has benefits over other methods. The simultaneous syringe technique has been successfully employed in more than 50 eyes, with both persistent and recurrent POVCH.

The simultaneous syringe technique is a single-stage procedure using a closed system. By having balanced injection and aspiration, it is the closest to replicating the conditions of PPV done in the OR. Compared to a two-needle open system [5], the contents of the vitreous cavity are collected in a syringe, with no risk of contaminating the field or the patient’s clothes. The sample can then be discarded cleanly or sent for lab analysis if desired.

A disadvantage of the one-needle open system techniques [7, 9] is IOP fluctuation with potential for extreme elevations in IOP. In the simultaneous syringe technique, this can be better managed with the two syringes. Due to the viscosity mismatch between the injected air and aspirated fluid, maintaining the intraocular pressure can be challenging. The eye is initially pressurized by injecting air prior to inserting the needle used for aspiration. The injecting provider digitally monitors the intra ocular pressure, and directs the assistant aspirating the blood to adjust the aspiration rate. While this creates some IOP fluctuation during the simultaneous syringe procedure, excessive high pressure is avoided by injecting air slowly and palpating the globe throughout the procedure.

Compared with using balanced saline for in-office vitreous cavity lavage [5], exchange with air is less expensive. As reported previously, office based AFX cost significantly less than going to the operating room for PPV, with calculated cost difference of about $2,121.45 USD [8]. Additionally, there may be less chance of endophthalmitis with an air-filled eye compared to a saline-filled eye, though more studies are needed [10].

The disadvantage of this technique is the difficulty of positioning both hands to safely hold the aspirating needle, while at the same time applying pressure on the injecting syringe. A second surgeon holding one of the needles is helpful while learning the technique, but with practice it is possible to control both needles as seen in the video.

In conclusion, the simultaneous syringe technique is an improved method of performing AFX for patients who are suitable candidates for an office-based procedure.

## Supplementary Information

**Additional file 1.** This video demonstrates the technique of office-based air fluid exchange using a two-needle, closed system with simultaneous air injection and fluid aspiration.

## Data Availability

Not applicable.

## Abbreviations

POVCH: Post-operative vitreous cavity hemorrhage
PPV: Pars plana vitrectomy
VEGF: Vascular endothelial growth factor
FVI: Fibrovascular ingrowth
AFX: Air-fluid exchange
IOP: Intraocular pressure
mm: Millimeters
mL: Milliliters
